# Falls When Standing, Falls When Walking: Different Mechanisms, Different Outcomes in Parkinson Disease

**DOI:** 10.7759/cureus.5329

**Published:** 2019-08-06

**Authors:** Abraham Lieberman, Aman Deep, Markey C Olson, Victoria Smith Hussain, Christopher W Frames, Margaret McCauley, Thurmon E Lockhart

**Affiliations:** 1 Neurology, Muhammad Ali Parkinson Center, Barrow Neurological Institute, Phoenix, USA; 2 Neurology, University of Tennessee Health Science Center, Memphis, USA; 3 Neurology, Muhammad Ali Parkinson Center, Bob & Renee Parsons Fall Prevention Center, Barrow Neurological Institute, Phoenix, USA; 4 Biomedical Engineering, Ira A. Fulton Schools of Engineering, Arizona State University, Tempe, USA

**Keywords:** falls when standing, falls when walking, locomotion, parkinson disease, postural stability

## Abstract

Our retrospective study of falls and resultant trauma in consecutive Parkinson disease (PD) patients seen in one year at the Muhammad Ali Parkinson Clinic found that multiple-fallers could be divided into patients who fell mainly when walking or those who fell mainly when standing. Patients who fell when walking were more likely to visit an emergency room or be admitted to a hospital.

Of 455 consecutive patients who were evaluated over a one-year period, 51 were excluded because they had atypical Parkinson disorders, had multiple risk factors for falling, or were demented. Unified Parkinson Disease Rating Scales and Zeno Walkway results were compared among non-fallers, single-fallers, and multiple-fallers. Among multiple-fallers, comparisons were made between patients who fell mainly when standing and those who fell mainly when walking.

Most patients (197, 49%) did not fall, 142 (35%) fell once, and 65 (16%) fell more than once. Multiple-fallers differed significantly from single-fallers and non-fallers: they had PD significantly longer (p<0.001), were more severely affected (p<0.001), and took shorter steps (p<0.001). Of 65 multiple-fallers, 26 (40%) fell mainly when standing, 28 (43%) fell mainly when walking, and 11 (17%) fell equally often when standing or walking. Falls when walking resulted in more severe injuries. Patients who fell mainly when standing did not realize they could fall when standing; engaged in inappropriate weight shifting, bending, reaching, and multitasking; and failed to use their assistive devices. Such patients would benefit from being counseled about falling when standing. Patients who fell mainly when walking were aware they could fall, despite using an assisted device, and were more likely to have freezing of gait (FOG). They were more likely to sustain a severe injury, and were more likely to be admitted to an emergency room or hospital. Such patients would benefit from reducing, if possible, FOG.

## Introduction

Parkinson disease (PD) is complicated by falls, which occur in 11% to 68% of patients [1-3]. Although falls in PD patients and in elderly patients often occur when the patients are standing or walking, the literature has not distinguished between them until recently [1-3]. Patients and many health care professionals are often unaware of these differences. We believe that falls when standing differ from those when walking in terms of severity of injury, response to levodopa, and possibly to deep brain stimulation (DBS) surgery.

The variability in reported fall prevalence (range, 11%-68%) is related to age; duration of PD; and inclusion or exclusion of patients with dementia, orthostatic hypotension, major orthopedic problems, or atypical PD [1, 2]. In a meta-analysis of six prospective studies of 473 patients, the fall rate was 46% and the best predictor of a future fall was two or more falls in the previous year [1]. Falls are a major cause of morbidity in PD patients, often resulting in serious injuries that require hospitalization [2, 4].

Falls are more likely to occur late in PD (>5 years after diagnosis). They are associated with higher (i.e., worse) scores on the Movement Disorder Society-sponsored revision of the Unified Parkinson’s Disease Rating Scale (MDS-UPDRS) [1-3, 5]. We studied the risk for falls in PD patients by reviewing the contributions of age and the duration and severity of PD [6-10]. In multiple-fallers, we compared the differences between patients who fell mainly when standing and those who fell mainly when walking.

## Materials and methods

This study was conducted in accordance with the recommendations of the Declaration of Helsinki for the study of human subjects. The Institutional Review Board at St. Joseph’s Hospital and Medical Center, Phoenix, Arizona, approved this retrospective study prior to the start of work. All patients consented in writing to the use of their information for research. No identifying patient information or images are included in this article.

Data were collected on 455 consecutive PD patients who were evaluated over a one-year period. Of the 455 patients who had idiopathic PD, as defined by established criteria [11], 51 were excluded because they had dementia (Mini-Mental State Examination score ≤24), blindness, orthostatic hypotension, neuropathy, or major orthopedic problems. These conditions were considered confounding risk factors for falls. Falls were defined as all four extremities, or the head, or the buttocks striking the ground.

All 404 patients were examined every three months for one year. Patients were instructed to keep “fall diaries” and to call or visit within 48 hours of any fall. In these follow-up visits, they were questioned as to whether they were standing or walking when they fell and whether they were “ON” levodopa (drug in therapeutic range) or “OFF” levodopa (drug not in therapeutic range). We asked multiple-fallers if they realized that they could fall when standing as well as when walking.

All patients were examined using the MDS-UPDRS Part III [12], including freezing of gait subtest (FOG, score 0-4) and postural stability subtest (“pull test,” score 0-4). Step length was evaluated using the Zeno Electronic Walkway (Zenometrics, LLC, Peekskill, NY, USA) and the ProtoKinetics Movement Analysis Software (PKMAS) (ProtoKinetics, LLC, Havertown, PA, USA). Patients who fell mainly when standing were asked if they were weight shifting (e.g., reaching, bending, stretching), if they were pushed, if they were multitasking, or if they were using an assistive device. Patients who fell mainly when walking were asked if they were turning or walking on an uneven surface, if they were pushed, or if they were using an assistive device. Multiple-fallers who fell mainly when standing were compared to multiple-fallers who fell mainly when walking, with consideration given to levodopa dosing, presence of FOG, presence of an abnormal pull test (a test of postural stability), and seriousness of injury from the fall.

## Results

The 404 patients were stratified into three groups: non-fallers (197 patients), single-fallers (142 patients), and multiple-fallers (65 patients). A one-way analysis of variance (ANOVA) was used to test for mean differences by group for the continuous variables of age, duration of PD, MDS-UPDRS score, and step length. Post hoc pairwise comparisons were conducted using independent-samples t-tests. The p value considered significant for the post hoc tests was adjusted to p<0.016 by taking the standard alpha of 0.05 and dividing it by the number of comparisons for each ANOVA (Table 1).

**Table 1:** Comparison of Non-fallers, Single-fallers, and Multiple-fallers FOG, freezing of gait; MDS-UPDRS, Movement Disorder Society-sponsored revision of the Unified Parkinson’s Disease Rating Scale; PD, Parkinson disease. *The pull test and FOG test were scored zero (normal) to four (worst). A one-way analysis of variance (ANOVA) was used to test for mean differences between patient groups for the continuous variables. Post hoc pairwise comparisons were conducted using independent samples t-tests. The P value considered significant for the post hoc tests was adjusted to p<0.016 by taking the standard alpha of 0.05 and dividing it by the number of comparisons for each ANOVA.

Variable	Non-fallers (n=197)	Single-fallers (n=142)	Multiple-fallers (n=65)	P Value	Single vs Non-fallers Post Hoc Comparison (P Value)	Multiple vs Single-fallers Post Hoc Comparison (P Value)	
Sex							
Male	106	74	33				
Female	91	68	32				
Age, years	66.0±6.1	68.3±6.2	70.1±6.8	0.001	0.04	<0.01	
PD duration, years	5.4±2.2	5.9±2.5	12.6±6.8	<0.001	0.05	<0.001	
MDS-UPDRS motor score	18.9±8.3	19.7±8.3	32.3±12.6	<0.001	0.38	<0.001	
Step length, meters	0.64±0.13	0.52±0.12	0.31±0.12	<0.001	<0.001	<0.001	
MDS-UPDRS Subtest	Abnormal Responses, No. (%)				Odds Ratio (95% CI) Post Hoc Comparison		
Pull test* >3	23 (12)	34 (24)	43 (66)	<0.001	2.4 (1.3, 4.3)		14.8 ( 7.5, 29.0)
FOG test* >1	12 (6)	19 (13)	31 (48)	<0.001	2.4 (6.6, 30.0)		14.1 ( 6.6, 30.0)

A similar analysis was applied to the 65 multiple-fallers who were divided into three groups: those who fell mainly when standing (26 patients), those who fell mainly when walking (28 patients), and those who fell equally when standing or walking (11 patients) (Table 2).

**Table 2:** Comparison of Multiple-fallers (n=65, 33 men, 32 women): Multiple-fallers Standing, Multiple-fallers Walking, and Multiple-fallers Standing and Walking FOG, freezing of gait; MDS-UPDRS, Movement Disorder Society-sponsored revision of the Unified Parkinson’s Disease Rating Scale; PD, Parkinson disease. *26 fell mainly while standing vs. 28 fell mainly while walking. †26 fell mainly while standing vs. 11 fell while standing and walking. ‡The pull test and FOG test were scored zero (normal) to four (worst). A one-way analysis of variance was used to test for mean differences between patient groups for the continuous variables.

Variable	Standing Falls (n=26)	Walking Falls (n=28)	Standing and Walking Falls (n=11)	Standing vs Walking Falls* P Value	Standing vs Both Standing and Walking Falls^†^ P Value	
Sex					≥0.05	
Male	13	15	5			
Female	13	13	6			
Age, years	72.0±7.1	68.4±6.6	69.9±6.3	≥0.05	≥0.05	
PD duration, years	13.1±1.2	12.2±1.1	12.5±1.2	≥0.05	≥0.05	
MDS-UPDRS motor score	36.4±14.6	28.6±11.2	30.4±13.1	≥0.05	≥0.05	
Step length, meters	0.30±0.13	0.31±0.14	0.33±0.13	≥0.05	≥0.05	
MDS-UPDRS Subtest Abnormal Responses, No. (%)						
Pull test^‡^ >3	19/26 (73)	18/28 (64)	6/11 (55)	≥0.05		≥0.05
FOG test^‡^ >1	12/31 (39)	15/31 (48)	4/11 (36)	≥0.05		≥0.05

The MDS-UPDRS subtests of FOG and postural control were analyzed for all 404 patients using a chi-squared cross-tabulation. Odds ratios are reported for the two-by-two post hoc analyses. There were three post hoc comparisons for each of the four motor subtests, totaling 12 post hoc comparisons (Table 1). Chi-squared tests were used to test for an overall difference in rates of abnormal test results among the three patient groups. Pairwise comparisons were then conducted with the smaller two-by-two contingency table, and odds ratios were reported using SPSS Statistics for Windows, version 21.0 (IBM Corp., Armonk, NY, USA) and Statistics Calculators version 3.0 (www.danielsoper.com).

Of the 404 patients, 197 (49%) did not fall, 142 (35%) fell once, and 65 (16%) fell more than once (Table 1). Age was a significant risk factor for falls, as was duration of PD (p≤0.001). Severity of PD, as assessed by the MDS-UPDRS motor score, was not significantly different between single-fallers and non-fallers (p=0.38). PD severity was worse (i.e., higher scores) in multiple-fallers than in single-fallers. Step length was significantly shorter in single-fallers than in non-fallers (p<0.001) and significantly shorter in multiple-fallers than in single-fallers (p<0.001) (Table 1).

Among the 65 multiple-fallers, 26 (40%) had a total of 104 falls and fell mainly when standing. Thirteen (50%) of the 26 patients fell only when standing: two fell when standing quietly and, to the best of our knowledge, were not orthostatic; three fell when trying to sit; five fell when pushed; 16 fell when they weight shifted while bending, stooping, reaching, or multitasking. Of the 26 patients, 20 (77%) were in an “ON” period (i.e., taking levodopa in a therapeutic range) when they fell; 20 (77%) had an abnormal pull test; eight (31%) had FOG; and 12 (46%) had an assistive device but were not using it when they fell. Only four (15%) of the 26 patients were aware that they could fall when standing. Six patients (23%) visited an emergency department, two more than once. Two patients were admitted to the hospital, one with a cervical cord injury and one with a concussion (transient loss of consciousness); both had a temporary worsening of their PD that lasted several months.

Of the 65 multiple-fallers, 28 (43%) had a total of 120 falls mainly when walking. Sixteen (57%) of these 28 patients fell only when walking. In four patients (14%), the falls were related to being pushed; in 17 patients (61%) the falls were related to turning or walking on an uneven surface. Fourteen patients (50%) were ON levodopa in a therapeutic range, and another 14 patients were OFF or going OFF levodopa and were not in a therapeutic range. Eleven patients (39%) had an abnormal pull test and 20 patients (71%) had FOG. Of 13 multiple-fallers (46%) who had an assistive device, only five patients were using it when they fell. Nine patients (32%) visited an emergency department (four at least twice) and four patients were admitted to a hospital; all these four patients had a concussion and they also experienced a temporary worsening of their PD.

Of the 65 multiple-fallers, 11 (17%) had a total of 40 falls and fell equally often when standing or walking. Five (45%) of these 11 patients had an abnormal pull test and four (36%) had FOG; five had an assistive device but only three were using it when they fell. Two patients who fell when walking visited an emergency room, and both were admitted to a hospital with a concussion and also experienced a temporary worsening of their PD.

Patients who fell mainly when standing were significantly more likely to have an abnormal pull test than those who fell mainly when walking (p=0.01; Fisher’s exact two-tailed test). There were no significant differences in the pull test results between patients who fell mainly when standing or walking and those who fell equally often when standing and walking.

Patients who fell mainly when standing were significantly more likely to be ON levodopa in a therapeutic range than those who fell mainly when walking (p=0.05). There were no significant differences in levodopa use between patients who fell mainly when standing or walking and those who fell equally often when standing and walking.

Patients who fell mainly when walking were significantly more likely to have FOG (p=0.01) than patients who fell mainly when standing. There were no significant differences in FOG between patients who fell mainly when walking or standing and those who fell equally often while walking or standing.

There were no significant differences in age, PD duration, UPDRS score, or step length between these three groups of multiple-fallers. Of the 26 patients who fell mainly when standing, 15 were female and 11 were male. Their mean age was 71.3±6.4 years, mean PD duration was 13.5±6.5 years, mean UPDRS score was 33.2±13.3, and mean step length was 0.28±0.11 meters. Of the 28 patients who fell mainly when walking, 12 were women and 16 were men. Their mean age was 68.5±6.5 years, mean PD duration was 11.3±5.7 years, mean UPDRS score was 30.5± 12.2, and mean step length was 0.32±0.13 meters. Of the 11 patients who fell equally often when standing and walking, six were women and five were men. Their mean age was 69.9±3.5 years, mean PD duration was 12.4±6.2 years, mean UPDRS score was 31.8±14.9, and mean step length was 0.30±12.8 meters.

## Discussion

The 65 multiple-fallers differed significantly from the single-fallers and non-fallers: they had PD significantly longer, were more severely affected, and took shorter steps (Table 1). Patients who fell once resembled non-fallers. This finding is not surprising because a single fall is as likely to result from an environmental hazard as it is from PD [13].

Until recently, investigators did not distinguish falls of patients when standing from falls of patients when walking. However, observational studies have documented some notable differences in these two types of falls. In 2014 Stevens et al. [14] reported on 328 persons who had 1,172 falls over five years. In 754 of the falls, the activity was specified: 497 (66%) of the falls occurred when walking and 257 of those (34%) occurred when standing. About the same time, in 2013, Robinovitch et al. [15] reported on their findings after videotaping 130 elderly people, none of whom had PD, over a 38-month period. These persons had a total of 227 falls. More than one-half of the falls occurred when the person was standing. Finally, in 2016, Weaver et al. [16] videotaped 306 persons, including 16 PD patients. PD patients had 71 falls, 1.3 times more than the non-PD patients. PD patients had more falls when they were standing than when they were walking. None of these reports commented on the importance of differentiating between falls when standing and those when walking.

In this study, we compared 26 multiple-fallers who fell mainly when standing with 28 multiple-fallers who fell mainly when walking. Twenty of the 26 multiple-fallers who fell mainly when standing were ON levodopa in a therapeutic range. Although patients who are ON are more likely to engage in activities that increase the risk of falling, we believe the falls resulted from impaired postural stability, an activity known to be unaffected by levodopa [17]. Postural stability, as assessed by the pull test, was significantly worse in multiple-fallers who fell mainly when standing. Additional levodopa did not reduce falls in these patients. Additional levodopa did, however, reduce falls in fallers who fell mainly when walking. Twelve multiple-fallers who fell mainly when standing had an assistive device, but none were using it when they fell. This finding indicates a lack of awareness in patients about the risk of falling when standing and reveals the need for more patient education and training.

Twenty of the 28 multiple-fallers who fell when walking had FOG compared to eight of the 26 multiple-fallers who fell mainly when standing. Numerous investigators have emphasized that FOG is a risk factor for falls [17]. FOG may or may not respond to levodopa.

Six of the 26 multiple-fallers who fell when standing visited an emergency department; two were hospitalized and had a temporary, several months’ long worsening of their PD. One had a concussion and one had a cervical spine injury - injuries that emphasize the need to examine both the cervical spine and the brain in patients who fall. Nine of the 28 patients who fell when walking visited an emergency department; four had a concussion and required hospitalization and also had a temporary worsening of their PD. Two of the 11 multiple-fallers who fell equally when standing or walking visited an emergency department. Both fell while walking, had concussions, and suffered temporary worsening of their PD. The finding of more severe injuries after falls when walking is not surprising, as falls when walking result in the patient striking the ground with greater force. There is much information on the possibility of head trauma causing PD [18]. There is less information on the effects of head trauma in PD patients. Goetz and Stebbins [19] studied 10 PD patients who sustained head trauma from motor vehicle accidents and reported a temporary worsening in their PD.

Postural stability and locomotion are linked. Postural stability, the ability to maintain one’s center of mass within a given base of support when standing, depends on the ability to integrate proprioceptive and vestibular input with muscular activity in the trunk and lower limbs [6, 19, 20]. Postural stability serves as “a brake”; it suggests the activation of “stop” neurons, as described by Bouvier et al. [21] We postulate that a defect in the activation of “stop” neurons is responsible for falls when standing. We further postulate that “stop” neurons may be cholinergic, noradrenergic, or GABAergic. Locomotion depends on the ability of the frontal and motor cortices to initiate and sustain movement and on the basal ganglia to relay this information to centers in the brainstem and spinal cord [10, 19, 20]. Locomotion suggests activation of “go” neurons, as described by Bouvier et al. [21] We postulate that a defect in the activation of “go” neurons is responsible for falls when walking. We postulate that “go” neurons may be dopaminergic. For the separate systems of postural stability and locomotion to function seamlessly and maintain an upright posture and normal gait, they must converge into a relatively confined space where the systems, although separate, are contiguous: where “stop” and “go” neurons are in proximity.

The pedunculopontine nuclei (PPN) of the brainstem fulfill these criteria, and the loss of PPN neurons has been linked to falls in PD patients [21-24]. The PPN is pivotal in scaling movement to proprioceptive input [21] and possibly in preventing falls.

Falls in PD occur when patients are standing as well as when they are walking. Such patients should be discouraged from “aggressive” weight shifting while standing, bending, reaching, and multitasking. Patients who fall when walking often have FOG. Patient education and training should emphasize helping patients to recognize situations that provoke FOG.

Falls when standing are not reduced by additional levodopa, which suggests the involvement of non-dopaminergic “stop” neurons. Patients who fall when standing may benefit from cholinomimetic [25-27] or noradrenergic drugs [28]. Patients who fall when walking may benefit from dopaminergic drugs.

DBS of the subthalamic nucleus or the globus pallidus interna reduces tremor, response fluctuations, and dyskinesias. However, DBS of the subthalamic nucleus or globus pallidus interna does not reduce falls and has a variable effect on FOG. DBS of the PPN can reduce both falls and FOG [29, 30]. The variability of DBS of the PPN in reducing falls and FOG may be related to not distinguishing falls when standing from falls when walking.

## Conclusions

In a study of 404 PD patients seen in one year, 65 patients (16%) fell more than once. Multiple-fallers had PD significantly longer and were more severely affected (higher UPDRS scores) than non-fallers or single-fallers. Among the 65 multiple-fallers, 28 fell mainly while walking and 26 fell mainly while standing. Patients who fell mainly while walking sustained more severe injuries. Patients who fell mainly while standing were unaware they could fall while standing and usually did not use an assistive device. Falls while standing occurred with patients who were “ON,” i.e., levodopa was therapeutic. Falls when walking usually occurred when patients were “OFF,” i.e., levodopa not in a therapeutic range. Falls while standing probably have a different mechanism from falls when walking. Patients and physicians should be aware of these differences.
